# Runoff, sediment, organic carbon, and nutrient loads from a Canadian prairie micro-watershed under climate variability and land management practices

**DOI:** 10.1007/s10661-023-11913-3

**Published:** 2023-10-09

**Authors:** Yongbo Liu, Nigel Van Nieuwenhuizen, Jane Elliott, Rajesh R. Shrestha, Ram Yerubandi

**Affiliations:** 1 Watershed Hydrology and Ecology Research Division, Environment and Climate Change Canada, Burlington, ON Canada; 2 Watershed Hydrology and Ecology Research Division, Environment and Climate Change Canada, Saskatoon, SK Canada; 3 Watershed Hydrology and Ecology Research Division, Environment and Climate Change Canada, Victoria, BC Canada

**Keywords:** Runoff, Water quality, Climate, BMPs, Spatio-temporal variation, Steppler subwatershed

## Abstract

This study conducted a spatio-temporal analysis of runoff, total suspended sediment, suspended particulate carbon, nitrogen, and phosphorus loadings within the 2.06 km^2^ Steppler subwatershed in southern Manitoba of Canada based on 11 years of field monitoring data collected at nine stations. Results showed that the nutrient losses were very small because of the implementation of multiple BMPs in the study area. However, a high spatio-temporal variation of runoff and water quality parameters was found for the nine fields within the subwatershed. The average runoff coefficient was 0.19 at the subwatershed outlet with sediment, suspended particulate carbon, total nitrogen, and total phosphorus losses of 73.8, 6.10, 4.54, and 0.76 kg/ha respectively. Spring snowmelt runoff was about 74.5% of the annual runoff at the subwatershed outlet, while for sediment, suspended particulate carbon, total nitrogen, and total phosphorus, the proportions were 61.1%, 63.6%, 74.9%, and 81.2% respectively during the monitoring period, which suggests that BMPs designed for reducing nutrient loadings from snowmelt runoff would be more effective than BMPs designed for reducing pollutant loading from rainfall storms in the study area. Research findings from this study will benefit the enhancement of current BMPs and the development of new BMPs in the region to minimize soil and nutrient losses from agricultural fields and improve water quality in receiving water bodies.

**Supplementary Information:**

The online version contains supplementary material available at 10.1007/s10661-023-11913-3.

## Introduction

Soil erosion and nutrient loss through agricultural runoff are major causes of land degradation and water quality deterioration in receiving water bodies in different regions across the world. This has become a particular concern in the Lake Winnipeg Basin (LWB) for which excessive nutrients, phosphorus (P) and nitrogen (N), loads from agricultural non-point sources and wastewater discharges have resulted in a decline in ecological conditions of Lake Winnipeg, the tenth largest freshwater lake in the world and the sixth largest in Canada (Rattan et al., 2017). The LWB covers nearly one million square kilometers, encompassing four Canadian provinces and four American states. The hydrological regime of LWB is dominated by spring snowmelt runoff, often occurring over frozen ground (Shrestha et al., 2021). In the southern Manitoba portion of LWB, a wide range of P export coefficient, from 0.01 to 1.58 kg/ha/yr, had been reported at multiple sampling stations along tributary streams and main rivers depending on conditions of climate, geology, soil, vegetation, and land use (Stewardship, 2011). Therefore, the study of runoff, sediment, and nutrient load variation in relation to climate and land management practices is essential to support the primary goals of reducing nutrient input from sources and improving lake water quality.

Agricultural water quality management in cold climate regions is more challenging and complex because of its unique agronomic, bio-geochemical, and hydrometeorological characteristics. This includes short growing seasons, limited water infiltration on frozen ground, vegetative nutrient release after freeze–thaw, and enhanced transport of late fall and winter nutrient applications, as well as the challenges that also exist in warm regions such as soil legacy nutrients and nutrient stratification (Liu et al., 2019a). Redistribution of snow by the wind results in irregular snow accumulation and snowmelt in spring, leading to an uneven distribution of runoff, sediment, and nutrient losses within a watershed (Costa et al., 2021). In addition to the climate effect, agricultural operations such as crop rotation, fertilizer, and manure application, tillage, irrigation, surface, and subsurface drains, and associated structural and non-structural best management practices (BMPs) also have a significant impact on nutrient export from crop fields (Bishop et al., 2005; Tiessen et al., 2010). These impacts complicate nutrient export processes at both field and watershed scales.

In the field of soil and water conservation, field studies are essential for gathering real-world information, identifying the main drivers of soil erosion and nutrient dynamics, and quantifying ecohydrological responses to climate and land use changes. However, short-term or event-based monitoring studies have a limited replication potential, resulting in less power and, therefore, potentially unreliable estimates when scaling up to a watershed level for a long-term assessment (Yang et al., 2022). Long-term field monitoring on a watershed scale can help to minimize the bias of low-frequency field studies and help to address runoff and pollutant delivery from individual land uses with a high temporal and spatial resolution (Remund et al., 2021). Therefore, long-term field studies in small watersheds are very valuable to better understand the mechanisms of runoff, sediment, and nutrient load variations in relation to climate and land management practices.

The South Tobacco Creek (STC) watershed in southern Manitoba, Canada was one of the pilot study watersheds in the Agriculture and Agri-Food Canada (AAFC) watershed evaluation of BMPs (WEBs) project from 2004 to 2012. Extensive field and modeling studies have been implemented in the study area over the past two decades based on long-term field monitoring data. Based on a paired field study within the STC, Tiessen et al. (2010), Liu et al. (2013), and Liu et al. (2014a) found that conservation tillage reduced the export of sediment and total N (TN) but increased dissolved P (DP) and total P (TP) with the snowmelt runoff the top driving factor affecting field‐scale losses of N and P in the study area. Liu et al. (2014b) concluded that annual crop and perennial forage had no effects on loadings of sediment, particulate N (PN), and particulate P (PP) under snowmelt runoff conditions but had significant effects on the dissolved N (DN) and DP based on 2005–2012 filed monitoring data collected at four experimental fields within the STC watershed. Based on data collected at two small dams within the STC watershed during 1999–2007, Tiessen et al. (2011) concluded that small dams were effective in reducing peak flows from agricultural land and could reduce sediment and nutrient loads significantly during both snowmelt and rainfall runoff. Li et al. (2011) studied the effects of multiple BMPs in two small subwatersheds within the STC watershed by comparing monitoring results between pre-BMPs and post-BMPs. Results showed that the collective reduction in runoff was mostly nonsignificant, but the implementation of these BMPs resulted in a significant reduction in nutrient losses from the treatment subwatersheds. Koiter et al. (2013) investigated the role of connectivity and scale in assessing the sources of sediment in the STC watershed using sediment source fingerprinting, while an economic analysis of agricultural BMPs in the STC watershed was implemented by Khakbazan et al. (2013). Chen et al. (2017) analyzed the changes in runoff chemistry and soil fertility after multiple years of cattle winter bale feeding on annual cropland based on 2007–2015 field monitoring data collected at two experimental sites within the STC watershed. The results showed that more runoff was produced from the bale feeding areas but with a lower nutrient concentration in comparison to feedlot sites. These earlier field studies focused on the effects of BMPs on runoff and nutrient losses from agricultural fields but lacked systematic analysis of their spatio-temporal variations in relation to climate and land management practices.

In addition to field studies, modeling attempts were also conducted in the STC watershed to assess the effects of small dams on stream flow and water quality, and sediment yield from upland and channel erosions using the Soil and Water Assessment Tool (SWAT) (Liu et al., 2014c, 2015). In a recent study, Wilson et al. (2019) analyzed surface soils as a source of P in snowmelt runoff from cropland based on 2013–2017 measurement data collected at 16 edge-of-field monitoring sites across the Red River Basin (RRB) and the Assiniboine River Basin (ARB) in Southern Manitoba. Results showed a clear link between near-surface soil P concentration and the potential of P loss with surface runoff for fields located in the LWB over a range of fertilizer management and tillage practices. Soil P was also the focus of a study by Liu et al. (2019a) based on 1997–2014 measurement data in the paired experimental fields within the STC watershed. Results showed that soil P drawdown by lowering fertilizer inputs had a large potential to reduce DP concentrations in both snowmelt runoff and rainfall runoff without affecting crop yields. A review by Liu et al. (2019b) on processes, drivers, management options, and research needs for agricultural water quality improvement in cold climates concluded that the nongrowing season had a critical role in annual nutrient losses, seasonal nutrient transport, and BMPs efficiency among years and across regions. An assessment of cross-region synthesis of edge-of-field results showed that snowmelt dominated runoff volume and P loss across Canada with varying drivers on P runoff pattern with most losses in a dissolved form in the Prairie region and most in particulate form in the Great Lakes region (Liu et al., 2021). These findings demonstrated a need for further understanding the interactions among water quality, climate, hydrology, land use, and land management in cold climate regions, which helps BMP management to reduce nutrient losses under the changing land use and climate variability.

This study aimed to better understand the spatio-temporal variation of runoff, sediment, and nutrient losses and their relations with climate, landscape features, and land management practices in the Steppler subwatershed (SSW) within the STC watershed based on eleven years (2005–2015) of field monitoring data. The specific objectives are (1) to quantify runoff, sediment, and losses of nutrient and suspended particulate carbon (SPC) from nine fields within the SSW, (2) to analyze their spatial distribution under different landscape and land management conditions, (3) to analyze their annual and seasonal variation during the monitoring period, and (4) to assess these losses with environmental variables and land management practices. Results from this study can provide site-specific information on runoff, sediment, and nutrient losses under Canadian prairie cold climate conditions and support policy development for the promotion of suitable agricultural BMPs on the non-point source (NPS) pollution control and their spatial management in the LWB.

## Materials and methods

### Study area

**Fig. 1:**
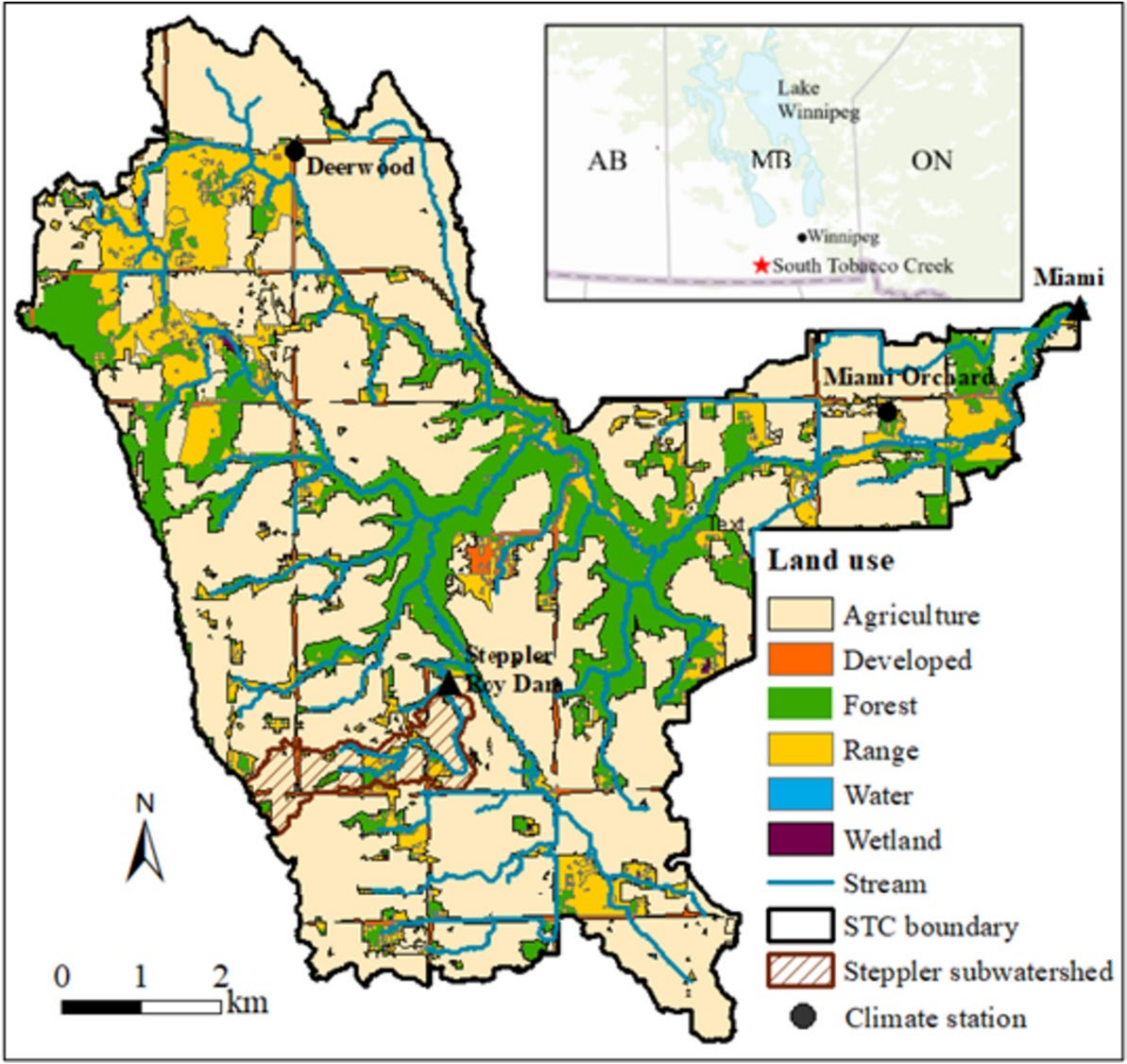
Land use and location of the study area

The SSW is a micro watershed (2.06 km^2^) located in the upstream portion of the STC watershed (75.0 km^2^) near the town of Miami within the drainage area of Lake Winnipeg in southern Manitoba of Canada (Fig. 1). The subwatershed is one of the case study areas in Li et al. (2011) for assessing the effects of multiple BMPs on hydrology and nutrient losses and in Liu et al. (2014a) for assessing nutrient and sediment losses in snowmelt runoff from perennial forage and annual cropland. Elevation in the study area ranges from 436 to 496 m (Electronic Supplementary Material (ESM), Figure S1a) with a mean slope of 3.56% based on a 10-m LiDAR-derived digital elevation model (DEM). High slopes are found on valley sides in up and downstream channels while low slopes are observed in upland fields (Figure S1b). The dominant soils in the study area are Dark Gray Chernozems formed on moderately to strongly calcareous glacial till with the texture of clay loam in the middle and downstream valleys and loam in upland fields (Figure S1c). Based on AAFC’s crop inventory data, agriculture (cereal crops, oilseeds, perennial forages, and livestock) is the dominant land use in the study area (72.6%), while other land uses are grasses (11.6%), forest (10.0%), road (3.89%), exposed land (1.05%) and water (0.87%) (Figure S1d).

The climate in the region can be classified as cool, continental semi-arid with a short warm summer and a long cold winter with a monthly average temperature below zero from November through March. Based on extended Livneh’s gridded climate data (1950–2015) over the study area (Livneh et al., 2015), the mean annual temperature is 2.52 °C ranging from -0.48 °C (1950) to 5.35 °C (1987), and the mean annual precipitation is 548 mm ranging from 339 mm (1988) to 776 mm (1954) with 25 to 30% occurring as snowfall. The growing season in the region is about five months from early May to early October. Both temperature and precipitation have a symmetric distribution with higher values in summer from June to August, and low values in winter. The average annual daily discharge measured at the STC outlet from 1964 to 2020 is 0.24 m/s (0.014–0.63) with a standard deviation of 0.17 m/s, and annual mean runoff of 67.5 mm (4.07 – 179 mm) with a standard deviation of 48.8 mm and an average runoff coefficient of 0.13. The majority of runoff occurs in spring from March to May (79.0%) with peaks in April or May due to snowmelt, while low flow occurs in the summer period with exceptions of multiday rainfall events or intense thunderstorms, and basically, there was no flow in the winter season from November to February.

**Fig. 2:**
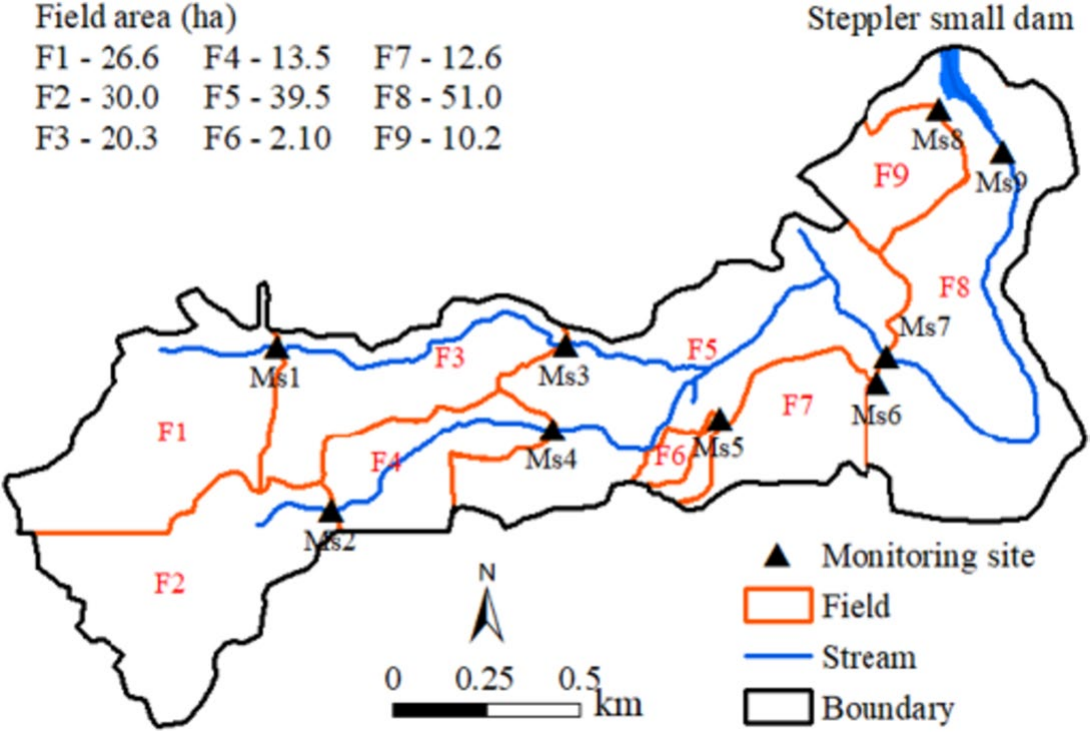
Fields and monitoring stations in the SSW

The Steppler Roy dam (Fig. 1) at the subwatershed outlet was constructed in 1987 with a storage capacity of 66.1 × 10^3^ m^3^ and a surface area of 2.30 ha (Liu et al., 2014a, 2014b, 2014c). The multipurpose small dam was designed to regulate seasonal storage, control spring and summer floodwater, and store water for summer uses. Most of the SSW is part of a single farm operation that has farm buildings and a cattle feedlot for overwintering/feeding with approximately 100 cows within the subwatershed (F6 in Fig. 2). The fields on the farm are separated by two small intermittent watercourses and are seeded with either cereal grains or oilseeds on a rotational basis (Fig. 2). Five BMPs including a holding pond downstream of the beef cattle yard, riparian and grassed waterway management, grazing restriction, forage conversion, and nutrient management were initiated in 2005 and designed to reduce nutrient loading from agricultural activities. These BMPs have altered the processes of runoff generation and transport and reduced nutrient losses to surface water significantly in the subwatershed (Li et al., 2011).

### Data collection

Flow and water quality have been monitored in the study area at the inlet of Steppler Roy reservoir (MS9 in Fig. 2) since 1999, the outlet of the cattle feedlot (MS5) since 2006, and other sites since 2005 (Table 1). V-notched weirs or circular flumes were installed to monitor runoff at the lower edge of the fields at MS5, MS6, and MS8, and the other six sites along the intermittent watercourses. The depth of water passing over the weirs or through the flumes was recorded at a 5-min interval and then converted to flow rate using a validated rating curve at each station. An automated water sampler controlled by a datalogger was installed at each weir or flume and took high-frequency samples during runoff events and supplementary grab samples during non-flooding periods. The procedures for the measurement of hydrological variables of runoff, water sampling, and laboratory analyses were detailed by Tiessen et al. (2010) and Liu et al. (2013). Flow monitoring and water quality sampling were conducted at each station from March to October covering all runoff events throughout the year. However, under specific circumstance, flow rate might be missed at the beginning of snowmelt or overestimated because of temporary ice block at the edge-of-field station during snowmelt period. These missing or overestimated flow data has been fixed in this study based on overall water balance of up or downstream stations. The drainage area, monitoring period, total number of runoff events, and water quality samples at each monitoring station are provided in Table 1.

**Table 1:** Drainage area, monitoring period, the total number of runoff events, and water quality samples at each monitoring station in the SSW

Station	Drainage area (ha)	Period (yr)	Events	Samples	Fields
MS1	26.6	2005–2015	76	299	F1
MS2	30.0	2005–2015	88	335	F2
MS3	46.9	2005–2015	90	362	F1, F3
MS4	43.5	2005–2015	74	319	F2, F4
MS5	2.10	2006–2015	102	409	F6
MS6	12.6	2005–2015	40	156	F7
MS7	132	2005–2015	82	493	F1, F2, F3, F4, F5, F6
MS8	10.2	2005–2015	56	186	F9
MS9	196	1999–2015	107	585	F1, F2, F3, F4, F5, F6, F7, F8

The Livneh’s 10-km gridded continuous daily climate data including precipitation and temperature (Livneh et al., 2015) at a station nearby the subwatershed was employed to assess its impact on runoff and pollutant loadings from each field during the sampling period from 2005 to 2015. Compared to observed station data at Deerwood and Miami Orchard, about 7.5 km in the north and northeast of the SSW (Fig. 1), the correlation coefficients of precipitation and temperature were higher than 0.9 over the data available periods which demonstrated the validity of Livneh’s climate data for analysis of runoff and pollutant loadings in this study.

A 1-m LiDAR-derived DEM, digitized subwatershed boundary, field boundary, and stream network were obtained from Prairie Farm Rehabilitation Administration (PFRA) of AAFC used for watershed and field delineation (Fig. 2). Soil characteristics in the SSW were analyzed based on soil data obtained from AAFC Soil Landscapes of Canada (SLC). In addition, 2005–2009 field land use survey data collected for the STC watershed during the WEBs project and AAFC’s 2010–2015 crop inventory data (30 × 30 m) were utilized in this study to analyze their impacts on runoff and pollutant loadings from each field. A summary of the dominant crop in each field during the period 2005–2015 is provided in Table 2. The majority of F6 is covered by a cattle feedlot and was not listed in the table. Forage (alfalfa), oilseed (canola and flax), and cereal (fall rye, spring wheat, and barley) are major crop types in the subwatershed during the sampling period.

**Table 2:** Characteristics of the nine fields, SSW and STC watershed

Field/ watershed	Area (ha)	Average elevation (m)	Average slope (%)	Dominant soil texture (%)		Dominant land use (%)	
F1	26.6	487	3.0	Loam	100	Crop	51.6
F2	30.0	489	4.0	Loam	100	Crop	80.1
F3	20.3	475	4.0	Loam	97.6	Crop	89.3
F4	13.5	476	7.0	Loam	100	Crop	66.8
F5	39.5	456	3.6	Loam	57.1	Crop	44.9
F6	2.10	457	5.3	Clay loam	99.3	Feedlot	40.0
F7	12.6	450	1.9	Loam	80.0	Crop	88.4
F8	51.0	444	2.9	Clay loam	53.1	Crop	79.3
F9	10.2	444	2.7	Loam	96.8	Crop	92.4
SSW	206	464	3.6	Loam	72.5	Crop	74.2
STC	7500	428	5.9	Loam	73.3	Crop	89.2

### Data analysis

Under cold climate conditions in the region, snowfall started in October or November each year, accumulated through the next March, and then melted in late March or April on frozen soils. To account for the amount of snow that accumulated from the previous year, annual runoff and pollutant loading estimates were conducted on a basis of the hydrological year (H-year) from September 1 to August 31 next year, while seasonal analyses were performed for spring (March–May), summer (June–August), autumn (September–November), and winter (December-February) from each field. Runoff and pollutant loading from F1, F2, F6, F7, and F9 were estimated based on measurements at station MS1, MS2, MS5, MS6, and MS9 directly, while F3, F4, F5, and F8 were calculated using MS3 subtracted by MS1 (MS3-MS1), MS4 subtracted by MS2 (MS4-MS2), MS7 subtracted by MS3 and MS4 (MS7-MS3-MS4), and MS9 subtracted by MS7 and MS6 (MS9-MS7-MS6) (Fig. 2). Flow measured at MS6 discharged to a holding pond right after the station was used for irrigation on neighboring fields and therefore was not subtracted from MS7 for the calculation of F7. MS9 is an inlet station of the Steppler Roy reservoir and does not cover side areas of the reservoir. In the runoff and loading analysis of F8, we assumed the same rate per unit area calculated from MS9-MS7-MS6 for the entire field.

Runoff (mm) at monitoring stations and from each field was calculated by the measured flow volume divided by the contributing area and was averaged at daily, monthly, seasonal and H-year scales. The annual runoff coefficient was calculated as the runoff divided by the H-year precipitation. Eight water quality parameters including TSS, SPC, PP, DP, TP, PN, DN, and TN were analyzed in this study. Because no relationships were found between discharge and pollutant concentrations at sampling stations, the linear interpolation method of actual sample concentrations between sampling times used by Bishop et al. (2005) and Tiessen et al. (2011) was applied to estimate pollutant concentrations at each time interval over a runoff event. During summer and fall low flow periods, grab samples were taken occasionally at different stations. For these low-intensity runoff events where only two samples were collected, pollutant concentration was averaged over the entire event for each parameter of interest. For runoff events where only one sample was available, that sample concentration value was assumed to be constant throughout the event. Pollutant loadings were calculated in kilograms as the product of flow volume and pollutant concentration and were summed for a given period and converted to kilograms per hectare by dividing pollutant loading during the period by the contributing area. The flow-weighted event-mean-concentration (EMC) of TSS, SPC, and nutrients was calculated by dividing the nutrient load by the total flow volume of the runoff event.

The temporal variation of runoff, erosion, and nutrient losses and their spatial variation among different fields were quantified by the standard deviation (SD), coefficient of variation (CV), and boxplot. SD is a measure of absolute variation that retains the units of measure, CV = SD/Mean is a measure of relative variability of observations with respect to the mean, while boxplot visually shows the distribution of observations and skewness through displaying the data quartiles and averages. The 11-year mean annual and seasonal values of each parameter were computed for each field to analyze the spatial patterns, and temporal variation during the sampling period from 2005 to 2015. To describe the relationship between a response variable (runoff, sediment, and nutrient loadings) and the influencing factor (climate, land use, and landscape features), a correlation analysis was performed to identify critical influencing factors evaluated by the coefficient of determination (R^2^) between each pair of parameters. In addition, the T-test *p*-value (probability associated with significance) was used to test the significance of the correlations at a 95% level, where a *p*-value < 0.05 is considered a significant correlation.

## Results

### Spatiotemporal variation of runoff

The annual precipitation (PCP), average temperature (TEM), runoff (R), and runoff coefficient (RC) of each field for each H-year during the monitoring period within the SSW are summarized in Table 3. Runoff from F8 was calculated by MS9-MS7-MS6 assuming the same average runoff depth in the 10-ha area downstream of MS9 before the SSW outlet. The runoff volume generated from the SSW was calculated by the sum of MS9, MS8, and the area downstream of MS9 before the SSW outlet. Because F6 is dominated by a beef cattle yard with significantly different runoff processes compared to other fields, it was not included in the SD and CV calculation in the table.

**Table 3:** Crops rotation during 2005–2015 within the SSW

Field/Year	F1	F2	F3	F4	F5	F7	F8	F9
2005	Canola	Barley	Flax	Alfalfa	Alfalfa	Alfalfa	Flax	Oats
2006	Wheat	Fall Rye	Wheat	Alfalfa	Alfalfa	Alfalfa	Wheat	Flax
2007	Canola	Canola	Canola	Alfalfa	Alfalfa	Alfalfa	Canola	Barley
2008	Wheat	Fall Rye	Wheat	Alfalfa	Alfalfa	Alfalfa	Wheat	Canola
2009	Fall Rye	Canola	Oats	Alfalfa	Alfalfa	Alfalfa	Flax	Oats
2010	Canola	Wheat	Canola	Canola	Canola	Wheat	Canola	Canola
2011	Cereals	Canola	Alfalfa	Cereal	Alfalfa	Alfalfa	Alfalfa	Alfalfa
2012	Canola	Cereals	Alfalfa	Canola	Cereals	Cereals	Canola	Alfalfa
2013	Wheat	Canola	Canola	Wheat	Alfalfa	Corn	Wheat	Wheat
2014	Canola	Flax	Oats	Canola	Wheat	Soybean	Canola	Canola
2015	Wheat	Canola	Canola	Wheat	Wheat	Corn	Wheat	Wheat

**Fig. 3:**
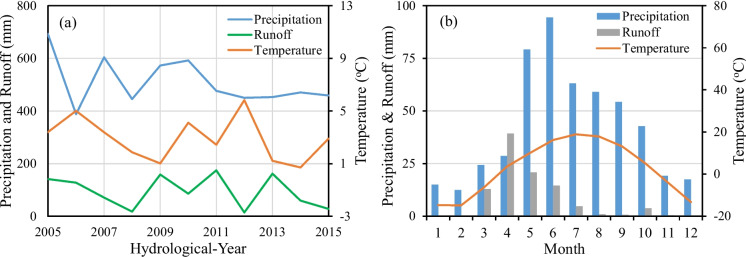
**a** Variation of annual precipitation, temperature, and runoff and **b** monthly distribution of precipitation, temperature, and runoff in the SSW during H-years 2005–2015

During the monitoring period, annual H-year precipitation varied considerably ranging from 388 to 693 mm with a mean of 510 mm, while annual H-year average temperature ranged from 0.71 °C to 5.84 °C with a mean of 2.90 °C. A significant H-yearly variation of runoff was also observed from each field during the monitoring period. (Fig. 3a). In general, high annual runoffs were observed in H-years with relatively high annual precipitation and low average temperature (e.g. 2009, 2011, and 2013) while low annual runoff was observed in H-years with relatively low precipitation and high average temperature (e.g. 2008, 2010, and 2012) affected by plant evapotranspiration and soil moisture content.

The temporal distribution of average monthly precipitation, temperature, and runoff in the SSW during the monitoring period was shown in Fig. 3b. June had the highest average precipitation followed by May and July while the winter period had the lowest average precipitation. Snowfall generally began in October and ends around May. The average annual snow was about 82.5 mm and was 16.2% of the average annual H-year precipitation. Temperature showed approximately a symmetric distribution with the highest average monthly temperature in July and the lowest in February. In contrast, runoff tended to a skewed symmetric distribution with the highest average runoff in April followed by May and June. Snowmelt and rain-on-snow runoff from March to May dominated the total annual runoff in the SSW during the monitoring period (Fig. 3a).

**Fig. 4:**
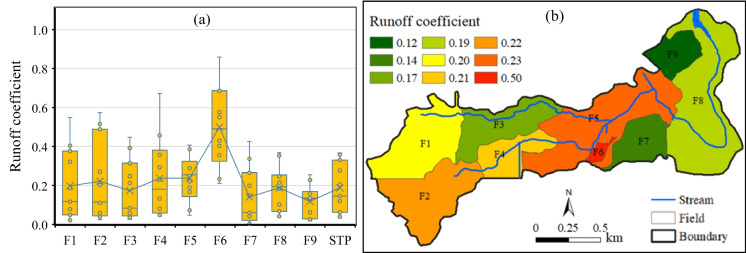
**a** Boxplot of field runoff coefficient and **b** spatial distribution of runoff coefficient during H-years 2005–2015 within the SSW

An annual H-year runoff coefficient Boxplot of the nine fields and the entire SSW is given in Fig. 4a. The Y-axis in the figure provides a measure of minimum, first quartile, mean (blue line), third quartile, and maximum runoff coefficient, which showed a high spatial variation among the nine fields during the 11-year monitoring period (Fig. 4b). The runoff coefficient calculated at the SSW outlet was 0.19 ranging from 0.03 to 0.37 among fields F1-F5 and F7-F9 excluding the cattle yard F6.

### Spatio-temporal variation of soil erosion and carbon losses

A summary of average seasonal and annual runoff, TSS yield, and SPC loss from each field and the SSW during the H-years 2005–2015 is provided in ESM Table S1. High yearly variations of TSS yield were observed from each field and the SSW during the monitoring period. The calculated annual TSS loading at the SSW outlet ranged from 5.33 to 288 kg/ha with a mean of 73.8 kg/ha. In general, high annual sediment yields were observed in H-years with high annual runoff (e.g. 2005 and 2013) while low annual sediment yield was observed in H-years with low annual runoff (e.g. 2012 and 2015) with R^2^ of 0.41 and T-test ρ-value of 0.18 between annual runoff and annual sediment loading at the SSW outlet, which were associated with runoff patterns and seasonal distributions.

**Fig. 5:**
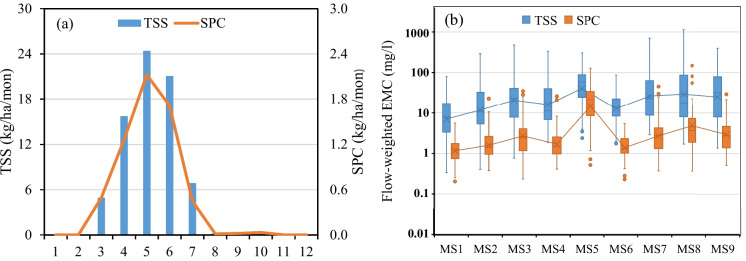
**a** Monthly distribution of average TSS and SPC yield from the SSW and **b** Boxplot of flow-weighted EMC during H-years 2005–2015

The monthly distribution of average TSS yield from SSW during the monitoring period 2005–2015 was shown in Fig. 5a. The average TSS yield from the SSW was about 45.1 kg/ha in spring, 28.1 kg/ha in summer, 0.56 kg/ha in autumn, and zero in winter (ESM Table S1). Unlike runoff, which peaked in April on all nine fields, some TSS yields peaked in April (F1, F2, F6, F7, F9), May (F3, F4), and June (F5, F8). The highest measured average monthly TSS yield occurred in May followed by June and April at the SSW outlet. The measured average monthly TSS concentrations at the SSW outlet were 57.2 and 70.6 mg/l respectively in May and June, higher than that in April (19.4 mg/l) due to more erosive rainfall and bare soil surface caused by crop planting and tillage activities. As a result, sediment yield was greater after snowmelt though May and June's runoff was less than April. This weakened the correlation between annual runoff volume and annual sediment yield in the SSW.

SPC had a very similar temporal distribution pattern as TSS. The calculated annual SPC loading at the SSW outlet ranged from 0.48 to 22.9 kg/ha with a mean of 6.10 kg/ha. High annual SPC yields were observed in H-years with high annual TSS yield (e.g. 2005 and 2013) while low annual SPC yield was observed in H-years with low annual TSS yield (e.g. 2008 and 2015). The monthly distribution of average SPC yield from SSW during the monitoring period 2005–2015 was shown in Fig. 5a. SPC yield from the SSW was about 3.88 kg/ha in spring, 2.17 kg/ha in summer, 0.054 kg/ha in autumn, and zero in winter (ESM Table S1). The highest average SPC yield occurred in May followed by June and April. Higher average monthly SPC concentration was measured in May and June at the SSW outlet compared to April.

Figure 5b gives a boxplot of flow-weight TSS and SPC EMCs at the nine sampling stations. A high temporal TSS and SPC variation at each sampling station and a high spatial TSS/SPC variation among the nine sampling stations were observed during the 11-year monitoring period. Maximum TSS and SPC flow-weighted EMCs were observed at station MS8 in May 2005 due to intense storms. Excluding MS5 (cattle yard station), mean TSS and SPC EMCs among MS1-MS4 and MS6-MS9 were 52.7/4.99 mg/l during the monitoring period, while TSS/SPC EMC ratios varied from 8.44 to 15.6 with an average of 11.5. MS5 had a different performance compared to other sampling stations with a high SPC EMC and a low TSS/SPC EMC ratio (2.66) due to cattle feeding activities. In general, flow-weighted TSS and SPC EMCs tended to increase from upland fields to downstream stations along the channel (Fig. 5b).

The spatial distribution patterns of average TSS and SPC yield at field scale within the SSW during the monitoring period 2005–2015 were similar (ESM Figures S2a and S2b). Lower TSS and SPC yields were found from upland fields (e.g. F1, F2, and F7), and higher TSS and SPC yields were found from fields along main channels (e.g. F3, F4, F5, and F8), and highest from F6 (cattle feeding yard). Overall, overland erosions from crop fields (F1-F5 and F7-F9) were very small ranging from 16.5 kg/ha to 138 kg/ha. These findings conformed the SWAT modeling result by Liu et al. (2015) that about 60% of annual sediment load was from channel erosion and 40% from overland at the STC watershed outlet based on sampling data collected during 1991–2010, and the cesium-137 tracer experimental result by Koiter et al. (2013) that most of the sediment originated from stream channels rather than upland fields at the STC watershed outlet.

### Spatio-temporal variation of P and N losses

A summary of average seasonal and annual PP, DP, and TP losses from each field and the SSW during the H-years 2005–2015 is provided in ESM Table S1. Similar to field runoff, TSS, and SPC, high yearly variations of P losses were observed from each field and the SSW during the monitoring period. The calculated annual PP/DP/TP loadings at the SSW outlet ranged from 0.005/0.050/0.055 to 0.44/1.17/1.61 kg/ha with a mean of 0.13/0.64/0.76 kg/ha. In general, high annual PP/DP/TP yields were observed in H-years with high annual runoff and TSS loading (e.g. 2005 and 2013) while low annual PP/DP/TP yields were observed in H-years with low annual runoff (e.g. 2012 and 2015). Annual PP/DP/TP loadings at the SSW outlet had an R^2^ of 0.34/0.82/0.72 and T-test ρ-value of 0.0002/0.0002/0.0002 with annual runoff, and an R^2^ of 0.95/0.70/0.82 and T-test ρ-value of 0.013/0.014/0.014 with annual TSS loading. Higher R^2^ was found between DP and runoff, and between PP and TSS.

**Fig. 6:**
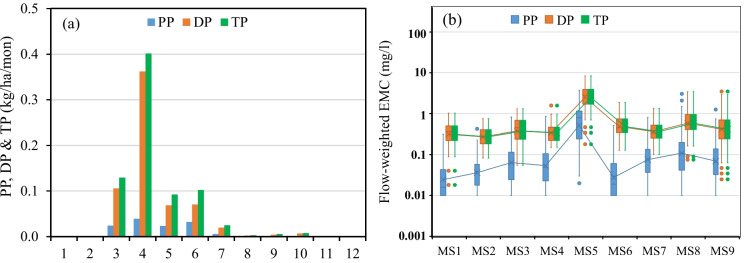
**a** Monthly distribution of average PP, DP, and TP yield from the SSW and **b** Boxplot of PP, DP, and TP EMC during H-years 2005–2015

The monthly distribution of average PP, DP and TP yield from SSW during the monitoring period was shown in Fig. 6a. PP, DP, and TP yield from the SSW was about 0.087, 0.54, and 0.62 kg/ha in spring, 0.038, 0.092, and 0.13 kg/ha in summer, 0.002, 0.012, and 0.014 kg/ha in autumn, and zero in winter (ESM Table S1). Monthly peaks of DP and TP yields from the nine fields were observed in April the same as runoff, while monthly peaks of PP yields occurred in the same month as TSS during the monitoring period. Higher monthly average PP concentration was found in May and June, while higher monthly average DP concentration was found in March and April, indicating that snowmelt runoff tended to cause more DP losses while rainfall storms tended to cause more PP losses from upland fields. This also caused a close correlation between annual runoff volume and annual DP yield and between annual TSS yield and annual PP yield in the SSW.

Figure 6b gives a boxplot of flow-weight PP, DP, and TP EMCs at the nine sampling stations. A high temporal PP/DP/TP variation at each sampling station and a high spatial PP/DP/TP variation among the nine sampling stations were observed during the monitoring period. DP was dominant in the TP EMC with an average DP/PP EMC ratio of 5.35 varying from 2.34 to 9.93 among the eight sampling stations excluding MS5. The cattle yard station MS5 had a lower average DP/PP ratio (3.95) due to the high TSS concentration and cattle feeding activities. In general, flow-weighted PP/DP/TP EMCs tended to increase from upland fields to downstream stations along the channel.

The spatial distribution of average PP and DP losses at the field scale within the SSW during the monitoring period is shown in EMS Figures S2a and S2b. PP losses had a similar spatial distribution pattern as TSS, while DP losses showed a different spatial distribution pattern compared to runoff and TSS yield. TP losses had the same spatial distribution pattern as DP because DP was dominant in the TP. Excluding F6, average annual PP losses from upland fields in the SSW was 0.13 kg/ha ranging from 0.05 kg/ha to 0.20 kg/ha. Average annual DP losses from upland fields in the SSW were 0.51 kg/ha ranging from 0.28 kg/ha to 1.05 kg/ha. Average annual TP losses from upland fields in the SSW were 0.64 kg/ha ranging from 0.44 kg/ha to 1.17 kg/ha. These PP, DP, and TP loadings are comparable and consistent with other field and modeling studies in the region (e.g. Liu et al., 2019a; Liu et al., 2013; and Li et al., 2011).

High yearly variations of N losses were observed from each field during the monitoring period (ESM Table S1). The calculated H-year PN/DN/TN loadings at the SSW outlet ranged from 0.03/0.26/0.29 to 2.43/8.54/10.5 kg/ha with a mean of 0.81/3.74/4.54 kg/ha. In general, high annual PN yields were observed in H-years with high annual TSS loadings while low annual PN/DN/TN yields were observed in H-years with low annual runoff. Annual PN/DN/TN loadings at the SSW outlet had an R^2^ of 0.60/0.89/0.87 and T-test ρ-value of 0.0002/0.0002/0.0002 with annual runoff, and R^2^ of 0.83/0.64/0.71 and T-test ρ-value of 0.014/0.015/0.015 with annual TSS loading. Higher R^2^ was found between DN and runoff, and between PN and TSS.

**Fig. 7:**
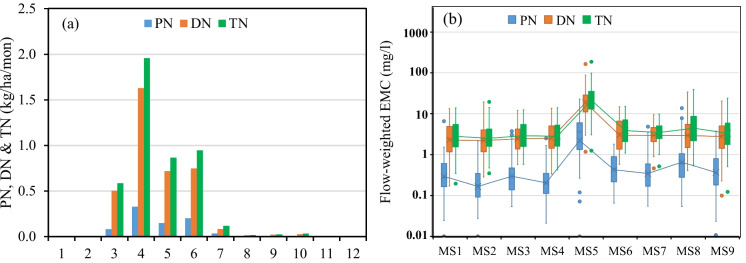
**a** Monthly distribution of average PN, DN, and TN loadings from the SSW and **b** Boxplot of PN, DN, and TN EMC during H-years 2014–2015

PN, DN, and TN yield from the SSW was about 0.56/2.85/3.40 kg/ha in spring, 0.24/0.84/1.08 kg/ha in summer, 0.011/0.047/0.058 kg/ha in autumn and zero in winter (ESM Table S1). Monthly peaks of DN and TN yields from the nine fields were observed in April the same as runoff, while monthly peaks of PN yields occurred in the same month as TSS during the monitoring period (Fig. 7a). A boxplot of flow-weight PN, DN, and TN EMCs at the nine sampling stations is shown in Fig. 7b. A high temporal PN/DN/TN variation at each sampling station and a high spatial PN/DN/TN variation among the nine sampling stations were observed during the monitoring period. DN was dominant in the TN EMC with an average DN/PN EMC ratio of 7.08 varying from 2.49 to 11.2 among the eight sampling stations excluding MS5. In general, flow-weighted PN/DN/TN EMCs tended to increase from upland fields to downstream stations along the channel.

The spatial distribution of average PN and DN losses at the field scale within the SSW during the monitoring period is shown in ESM Figures S4a and S4b. PN losses had a similar spatial distribution pattern as TSS, while DN losses showed a different spatial distribution pattern compared to runoff and TSS yield. Excluding F6, average annual PN/DN/TN losses from upland fields in the SSW were 0.81/3.90/4.71 kg/ha ranging from 0.27/3.03/3.30 kg/ha to 0.89/8.52/9.42 kg/ha (ESM Table S1). The average annual TSS/TN/TP ratio calculated at the SSW outlet was 97/6/1. These results are comparable and consistent with other field and modeling studies in the region (e.g. Rattan et al., 2017; Liu et al., 2014b; and Li et al., 2011).

## Discussion

### Impact of climate variability

Precipitation and temperature have a direct impact on annual runoff during the monitoring period in the SSW (Fig. 3a). High annual precipitation with low annual temperature resulted in a high annual runoff, while low precipitation with high temperature resulted in a low annual runoff. Because the majority of annual runoff comes from snowmelt in the area, the amount of snow accumulated during the winter period and the temperature in spring during the snowmelt period play a vital role in annual runoff and spring flooding. Two examples are H-year 2009 for which the total snow water depth was 112 mm and spring temperature was 1.2 °C, and H-year 2011 for which the total snow water depth was 87.6 mm) and spring temperature was 5.9 °C. The high snow water depth with moderate spring temperature in the H-year 2009 and the high spring temperature with moderate snow water depth in the H-year 2011 caused severe floodings in the spring and high annual runoff in the H-year 2009 and 2011 (Fig. 3a). On the contrary, in the H-year 2012, the total snow water depth was 34.5 mm and the spring temperature was 1.3 °C. The low snowpack and low spring temperature caused a small spring flow and annual runoff in 2012 at the subwatershed outlet.

Based on the analysis in this study, loadings of TSS, SPC, PP, and PN are associated with both summer rainfall runoff and spring snowmelt runoff in the SSW, while DP and DN are mainly controlled by snowmelt runoff due to nutrient releasing from plant residues during the freezing and thaw process which support earlier studies by Liu et al. (2013). Because DP and DN dominate field TP and TN exports in the study area, the amount and pattern of spring snowmelt runoff are critical to controlling TN and TP loadings at the subwatershed outlet. This is evident in H-years 2009 and 2011 during which high annual TP and TN yields were observed due to the high spring snowmelt runoff. Conversely, in H-year 2012, the annual TP and TN yields were very small at the subbasin outlet because of the small snowmelt runoff in spring. These findings reveal that BMPs designed for reducing nutrient loadings from snowmelt runoff, such as holding ponds, small dams, wetland restoration, and nutrient management, would be more effective than BMPs that rely on vegetation to protect from erosion to reduce pollutant loading from rainfall storms in the study area.

In a modeling study of snowpack response to climate change in the ARB-RRB by Shrestha et al. (2021), observed climate data during a recent period showed that there has been a consistently increasing trend in the minimum annual air temperature in the region while no significant trend in annual precipitation and maximum temperature. Seasonally, winter precipitation exhibited a slightly increasing trend and spring air temperature exhibited a significantly increasing trend in the RRB. Future climate projections of eight statistically downscaled global climate models (GCMs) indicated that the majority of GCMs projected future wetter winters and springs, and drier summers with an increased temperature leading to higher rainfall-to-snowfall ratio compared to the 1986–2005 baseline period. The changes would have major implications on hydrologic regimes in the region. Particularly mid-winter snowmelt, rain-on-snow events, and heavy rainfall events during and after snowmelt could impact flood generation processes and affect nutrient fluxes. Furthermore, the projected future streamflow changes in the Assiniboine basin by Dibike et al. (2021) revealed increased spring flows and earlier timings of peak spring flow. Such changes in the magnitude and seasonality of spring flow could also lead to increased spring nutrient loadings under future climate change conditions.

### Impact of landscape features

The SSW is dominated by loamy soils across the nine fields, therefore, the impact of soil type on runoff, sediment, and nutrient loadings at the field scale was not assessed in this study. The average surface slope of the subwatershed is 3.6% ranging from 1.9% to 7.0% (Table 4). A positive non-significant relationship was found between slope and normalized annual runoff fraction (field runoff divided by the subwatershed runoff) and between slope and normalized TSS yield fraction (field TSS yield divided by subwatershed TSS yield) based on a correlation analysis of the eight crop field results. The normalized PP and PN loading fractions also showed a positive non-significant relationship with surface slope. However, because DP and DN are dominant in TP and TN, positive relationships between slope, TP, and TN loadings were not obtained based on the correlation analysis, which is different from other field studies with high surface slopes (e.g. Ramos et al., 2019 and Zhang et al., 2020). This indicated that in the study area with a low slope gradient (< 7%) and under cold climate conditions, other than slope, factors such as land use, grazing activity in the riparian area, and land management practices play a more important role in TP and TN loadings from each crop field and at the subwatershed outlet.

**Table 4:** Annual precipitation, average temperature, runoff depth, and runoff coefficient of each field during H-years 2005–2015 within the SSW

H-year	PCP	TEM	R1	R2	R3	R4	R5	R6	R7	R8	R9	SSW	SD	CV
	(mm)	(^o^C)	(mm)	(mm)	(mm)	(mm)	(mm)	(mm)	(mm)	(mm)	(mm)	(mm)	(mm)	
2005	693	3.40	223	188	172	159	132	279	138	149	103	142	36.8	0.26
2006	388	5.02	146	200	122	138	117	266	165	137	88.6	128	33.3	0.26
2007	604	3.39	70.8	69	35.2	49.0	134	214	36.5	61.8	37.4	71.7	32.6	0.46
2008	445	1.87	8.41	26.1	15.5	21.5	32.4	382	0.33	18.6	11.5	18.1	10.2	0.56
2009	573	1.02	107	116	122	168	221	330	84.6	211	146	159	49.4	0.31
2010	592	4.11	46.2	65.5	49.6	78.5	98.6	409	29.5	146	100	86.1	37.5	0.44
2011	477	2.44	262	233	187	218	194	101	127	87.8	76.0	175	72.2	0.41
2012	450	5.84	9.48	17.7	11.7	19.1	21.2	248	1.61	18.0	7.82	14.7	6.78	0.46
2013	453	1.22	183	260	203	305	137	195	152	115	61.2	162	78.8	0.49
2014	471	0.71	23	20.4	21.5	29.3	136	110	9.63	91.4	34.7	60.1	44.2	0.74
2015	459	2.91	27.4	11.2	19.8	12.0	58.9	-	19.3	30.9	12.9	28.5	15.8	0.55
Mean	510	2.90	101	110	87.3	109	117	253	69.4	97.0	61.7	97.9	19.8	0.20
SD	91.8	1.66	90.5	94.3	75.4	96.5	61.7	103	65.1	61.8	45.1	60.8	18.6	0.31
CV	0.18	0.57	0.90	0.86	0.86	0.89	0.53	0.41	0.94	0.64	0.73	0.64	0.15	0.23
RC			0.20	0.22	0.17	0.21	0.23	0.50	0.14	0.19	0.12	0.19	0.04	0.21

A negative relationship was found between cropland area proportion and annual runoff with an R^2^ of 0.56. One of the reasons is that snow would redistribute from cropland to riparian non-crop areas and local depressions outside of the field due to strong wind during the winter period resulting in less snowmelt runoff in spring and less snowmelt runoff from the field during spring period (Van Hoy et al., 2020). For example, F5 has the lowest cropland area proportion (44.9%) but the highest runoff coefficient (0.23), while F9 has the highest cropland area proportion but the lowest runoff coefficient (0.12) among the eight cropland fields within the SSW. However, no relationships were found between cropland area proportion and TSS, TP, and TN loadings of the eight crop fields within the SSW.. This is because a lot of noises outside of cropland area were included in the field-averaged analysis in the SSW causing difficulties to find the relationship between cropland area proportion and pollutant loadings. An additional analysis by looking at different crop types in each field and evaluating the results on an annual basis is required to quantify the effect of cropland area proportion on pollutant loadings with this dataset.

The main crop types were canola, forage, spring wheat, oat, flax, and barley in the SSW during the monitoring period (Table 2). Crop management practices such as seeding, harvest, fertilizer and manure application, tillage, and residual management with respect to different crop types would have a significant impact on runoff, TSS, TP, and TN yield from each field. The effects of crop management practices on TP and TN yields can be demonstrated by comparing F1 (slope of 3.0%, loam, and 51.6% of cropland) with F2 (slope of 4.0%, loam, and 80.1% of cropland), where F1 had more canola and spring wheat years with higher N and P application rates and more intensive tillage compared to F2 during the monitoring period (Table 2). The observed F1 average annual runoff (101 mm) and soil loss (16.5 kg/ha) were less than the F2 annual runoff (110 mm) and soil loss (22.4 kg/ha) (ESM Table S1). However, the observed F1 average TP (0.56 kg/ha) and TN (4.46 kg/ha) loadings were higher than F2 average TP (0.44 kg/ha) and TN (3.30 kg/ha) loadings. More discussions about the impacts of crop management practices on runoff, sediment and nutrient losses in the study area can be found in Liu et al. (2014a) and Liu et al. (2013).

### Impact of land management practices

Five BMPs were implemented in the SSW (Table 5) in the WEBs project in 2006. The objective was to monitor their effect on nutrient concentrations and loadings within the watershed, their effect on flow volumes, and the cumulative effect of multiple BMPs at the watershed outlet (AAFC, 2012). The BMP of manure holding pond downstream of a confined cattle feedlot (F6) and the conversion of annual cropland to forage (F9) were monitored individually, while the impacts of other three BMPs, including riparian area management, grazing management, and nutrient management were monitored collectively at stations along the stream channel. Before the WEBs project, runoff and water quality were monitored at the outlet of SSW from 1999. The collective impacts of BMPs can be analyzed by comparing the monitoring results at the SSW outlet before and after BMPs implementation.

**Table 5:** Field BMPs and farming practices in the SSW

Field	Treatment	Field	Treatment
F1 F2	Annual crop rotation Annual crop rotation	F6	Feedlot area Runoff was captured by a holding pond
F3	Converted crop to forage, rotational grazing in the riparian area, no fertilizer application when in forage	F7	Annual crop rotation, fertilizer application based on soil testing
F4	Converted crop to forage, rotational grazing in the riparian area, fertilization based on soil testing	F8	Annual crop rotation widened riparian area with buffer harvesting
F5	Rotational grazing, no grazing on pastureland after mid-August	F9	Converted annual crop to forage, no fertilizer application when in forage

Based on the analysis of 65 snowmelt and rainfall runoff events for seven years (1999–2005) before BMPs implementation and three years (2006–2008) after BMPs implementation, Li et al. (2011) concluded that the collective impacts of the five BMPs were mostly nonsignificant on hydrology, but very significant on reduction of nutrient losses in the treatment subwatershed. Compared to the monitoring results before the WEBs project, the five BMPs collectively reduced the average annual TP, DP, and PP export by 38%, 41%, and 42%, and the average annual TN, DN, and PN export was reduced by 41%, 43%, and 38%, respectively at subwatershed outlet. In particular, the manure holding pond alone could reduce TP and TN loading on average at the subwatershed outlet by 17.3% and 18.9% based on numbers in ESM Table S1 of this study and reach a maximum TP and TN reduction by 64% and 57% respectively for a specific runoff event (Li et al., 2011). The nutrient budget analysis revealed that N inputs were reduced by 36% and P inputs were reduced by 59% in the SSW following the implementation of nutrient management strategies, which resulted from lower fertilizer application rates on annual cropland and minimal fertilizer applications on the land converted to perennial forage. However, no significant difference was found in the annual crop yields compared to pre-BMPs largely due to crop uptake of nutrients from prior applications (Khakbazan, 2013). These studies demonstrated that the combination of multiple BMPs in the SSW can reduce nutrient losses effectively from agricultural lands into water bodies. Therefore, the analyzed P and N exports from each treatment field and at the subwatershed outlet in this study would be smaller than other similar agricultural watersheds but with conventional land management practices. More discussions about the impacts of land management practices on runoff and water quality in the study area can be found in Li et al. (2011) and Khakbazan et al. (2013).

### Uncertainty and future work

The spatial and temporal variation analysis in this study was conducted based on monitored flow and water quality data collected at the nine field and stream stations within the SSW. The continuous water level was automatically recorded at each monitoring station. However, the V-notched weirs at the monitoring sites might be blocked by floating ice in early spring snowmelt events or by plant debris in summer storm events, which could result in an overestimation of flow rate at the station and overestimation of runoff volume and pollutant export from the field. Although dense automatic samples were taken at each station during a high flow period, some low flow events were grab sampled with a low frequency, which might not capture the event concentration variation and could result in an over or underestimate of the flow-weighted EMC and associated sediment and nutrient loadings of the event. Furthermore, the relationship between water quality parameters and climate, surface slope, land use, and land management in this study was obtained based on analyzing the results of eight experimental agricultural fields (excluding F6) with a relatively uniform soil type. It may not cover all types of upland hydrological and water quality processes in the region and may not represent relationships in areas with different climates, landscapes, land uses, and land management practices.

The spatial and temporal variation of field runoff, SPC, TSS, TN, and TP losses within an agricultural watershed are affected by many influencing factors. In addition to the factors of climate, slope, land use, and land management practices that were analyzed in this study, other factors such as wetlands and depressions (Baulch et al., 2021), small dams (Tiessen et al., 2011), connectivity between hillslopes and the river channel (Koiter et al., 2013), soil N and P concentration (Liu et al., 2019a), and stratification of nutrients at the soil surface due to reduced mixing of fertilizers or manure by tillage (Liu et al., 2013) also have significant impacts on runoff and water quality processes in the region at both field and watershed scale. The multi-influencing factors would make the water and nutrient cycle a very complex system and add more uncertainties to the temporal and spatial analysis of runoff and water quality parameters in this study. The measured TSS, SPC, and nutrient export rates from the 2.06 km^2^ SSW were much smaller than the export rates measured at the 75 km^2^ STC watershed outlet (Fig. 1) over the monitoring period, which demonstrated the effects of multiple BMPs on on-site reduction of TSS, SPC, and nutrient export from the SSW. However, off-site effects of field BMPs on TSS, SPC, and nutrient load reduction at a larger watershed scale remained uncertain from this study.

High-frequency flow and water quality measurements in the Steppler and some other subwatersheds within the STC watershed during the WEBs project have formed a basis for a detailed study of runoff and nutrient cycle dynamics under Canadian prairie cold climate conditions. Building on these analyses, future work may address questions such as (1) spatio-temporal variation of runoff and water quality at a watershed scale under existing conditions, (2) the effectiveness of various individual BMPs on nutrient load reduction under different climates, landscapes, soils, and land use conditions, (3) effectiveness of multiple BMPs on nutrient load reduction at watershed scale under baseline conditions, and (4) scaling-up of the monitoring results by combining with watershed water quality models and quantify the off-site effects of individual and multiple BMPs on TSS and nutrient load reductions at a watershed scale.

## Conclusion

This study conducted a spatial–temporal analysis of runoff, TSS, SPC, N, and P loadings in a Canadian prairie micro-watershed under climate variability and land management practices. Results from 11 years (2005–2015) of field runoff and water quality data collected at nine monitoring stations in the SSW in Southern Manitoba were analyzed in relation to climate, landscape features, land use, and land management practices. The annual analysis in this study was performed on a H-year basis (September–August) to account for the effect of snow accumulation on spring snowmelt runoff and pollutant loadings, and factors influencing the seasonality of runoff and pollutant loadings were also investigated.

Due to the implementation of multiple BMPs in the study subwatershed, the overall TSS, SPC, and nutrient export rates were much smaller compared to observations in other agricultural fields with conventional land management practices. Runoff exhibited a high spatial variation with a mean runoff coefficient of 0.19 ranging from 0.12 to 0.50 among the nine fields and a high temporal variation with a mean annual runoff depth of 97.9 mm ranging from 14.7 mm to 175 mm at the subwatershed outlet during the monitoring period. Similar spatial and temporal patterns were found for TSS, SPC, PP, DP, TP, PN, DN, and TN flow-weighted EMCs and loadings in the study area. The calculated average annual TSS export from the subwatershed was 73.7 kg/ha, SPC 6.11 kg/ha, PP 0.13 kg/ha, DP 0.51 kg/ha, TP 0.64 kg/ha, PN 0.81 kg/ha, DN 3.90 kg/ha, and TN 4.71 kg/ha with an average TSS/TN/TP ratio of 97/6/1. Spring snowmelt runoff at the subwatershed outlet was about 74.5% of the annual H-year runoff, while for TSS, SPC, PP, DP, TP, PN, DN, and TN, the proportions were 61.1%, 63.6%, 68.5%, 83.9%, 81.2%, 69.1%, 76.3%, and 74.9% respectively during the monitoring period. DP, TP, DN, and TP were associated more with spring snowmelt runoff, while TSS, SPC, PP, and PN were associated with both spring snowmelt runoff and summer rainfall storm runoff. This suggested that BMPs targeted at reducing nutrient loadings from snowmelt runoff would be more effective than BMPs designed for reducing pollutant loading from rainfall storms in the study area.

Spatial analysis showed that TSS and nutrient loadings did not have a clear relationship with surface slope but had a close relation with land use and land management practices in the study area. There is a high potential for increased soil and nutrient losses from upland fields under future climate change conditions if winter snow and spring temperature in the region increase. Therefore, the implementation of BMPs is essential in the region for environmental protection and sustainable development. Based on the field monitoring data collected in the WEBs project, future work should focus on the study of the spatio-temporal variation of water quality processes at a watershed scale and the effectiveness of various individual and multiple BMPs on nutrient load reduction under different climate, landscape, soil, and land use conditions. These research findings will benefit the enhancement of current BMPs and the development of new BMPs in the region to minimize soil and nutrient losses from agricultural fields and improve water quality in receiving water bodies.

### Supplementary Information

Below is the link to the electronic supplementary material.

Supplementary file1 (DOCX 1044 KB)

## Data Availability

The flow and water quality data (2005–2015) used in this study were collected during the AAFC’s WEBs project. The climate data used for analysis were extracted from the Livneh gridded climate dataset.
