# Supplementary material for: Runoff, sediment, organic carbon, and nutrient loads from a Canadian prairie micro-watershed under climate variability and land management practices
Source: Environ Monit Assess. 2023 Oct 9;195(11):1285. doi: 10.1007/s10661-023-11913-3 (PMC10562304; doi:10.1007/s10661-023-11913-3)
Supplement: Supplementary file 1 — Supplementary file1 (DOCX 1044 KB) [file 10661_2023_11913_MOESM1_ESM.docx]

Supplemental Material:

**Runoff, sediment, organic carbon, and nutrient loads from a Canadian prairie micro-watershed under climate variability and land management practices**

Yongbo Liu^a,^*, Nigel VanNieuwenhuizen^a^, Jane Elliott^b^, Rajesh Shrestha^c^, Ram Yerubandi^a^

^a^ Watershed Hydrology and Ecology Research Division, Environment and Climate Change Canada, Burlington, ON, Canada

^a^ Watershed Hydrology and Ecology Research Division, Environment and Climate Change Canada, Saskatoon, SK, Canada

^c^ Watershed Hydrology and Ecology Research Division, Environment and Climate Change Canada, Victoria, BC, Canada

Figure S1. Elevation (a), slope (b), soil texture (c) and land use (d) in the SSW

Table S1. Average seasonal and annual R (mm) and TSS, SPC, PP, DP, TP, PN, DN, TN yield (kg/ha) from each field during H-years 2005-2015 within the SSW

| Season | Parameter | F1 | F2 | F3 | F4 | F5 | F6 | F7 | F8 | F9 | SSW | SD | CV |
| --- | --- | --- | --- | --- | --- | --- | --- | --- | --- | --- | --- | --- | --- |
| Spring | R | 75.2 | 83.9 | 69.7 | 88.7 | 84.3 | 184 | 57.2 | 72.7 | 54.2 | 72.9 | 12.4 | 0.17 |
|  | TSS | 10.6 | 17.0 | 78.6 | 76.7 | 22.5 | 163 | 22.3 | 23.6 | 31.0 | 45.1 | 26.8 | 45.1 |
|  | SPC | 1.14 | 1.70 | 6.07 | 5.81 | 2.35 | 61.0 | 2.41 | 3.52 | 4.51 | 3.88 | 1.86 | 3.88 |
|  | PP | 0.045 | 0.037 | 0.11 | 0.14 | 0.11 | 1.95 | 0.088 | 0.070 | 0.18 | 0.087 | 0.047 | 0.087 |
|  | DP | 0.37 | 0.36 | 0.26 | 0.42 | 0.36 | 5.82 | 0.78 | 0.92 | 0.60 | 0.54 | 0.23 | 0.54 |
|  | TP | 0.41 | 0.40 | 0.36 | 0.56 | 0.46 | 7.77 | 0.86 | 0.99 | 0.78 | 0.62 | 0.24 | 0.62 |
|  | PN | 0.27 | 0.22 | 0.50 | 0.56 | 0.46 | 10.5 | 0.58 | 0.86 | 0.57 | 0.56 | 0.20 | 0.56 |
|  | DN | 3.28 | 2.67 | 1.71 | 2.66 | 3.04 | 59.1 | 6.92 | 1.67 | 3.74 | 2.85 | 1.66 | 2.85 |
|  | TN | 3.55 | 2.88 | 2.21 | 3.22 | 3.50 | 69.6 | 7.49 | 2.53 | 4.31 | 3.40 | 1.66 | 3.40 |
| Summer | R | 21.3 | 20.3 | 14.1 | 17.0 | 24.1 | 50.0 | 11.8 | 20.6 | 7.22 | 20.2 | 5.66 | 0.28 |
|  | TSS | 5.72 | 4.93 | 59.3 | 48.4 | 19.3 | 28.8 | 12.1 | 37.3 | 9.8 | 28.1 | 21.0 | 28.1 |
|  | SPC | 0.48 | 0.43 | 4.99 | 3.18 | 1.57 | 11.2 | 1.00 | 2.58 | 1.29 | 2.17 | 1.56 | 2.17 |
|  | PP | 0.014 | 0.009 | 0.081 | 0.076 | 0.029 | 0.40 | 0.029 | 0.053 | 0.020 | 0.038 | 0.028 | 0.038 |
|  | DP | 0.13 | 0.034 | 0.030 | 0.12 | 0.093 | 1.92 | 0.26 | 0.093 | 0.050 | 0.092 | 0.08 | 0.092 |
|  | TP | 0.14 | 0.043 | 0.11 | 0.20 | 0.12 | 2.33 | 0.29 | 0.15 | 0.070 | 0.13 | 0.08 | 0.13 |
|  | PN | 0.14 | 0.046 | 0.42 | 0.26 | 0.17 | 1.94 | 0.30 | 0.24 | 0.12 | 0.24 | 0.12 | 0.24 |
|  | DN | 0.72 | 0.34 | 0.42 | 0.81 | 0.77 | 13.1 | 1.52 | 1.44 | 0.070 | 0.84 | 0.51 | 0.84 |
|  | TN | 0.87 | 0.39 | 0.84 | 1.07 | 0.94 | 15.0 | 1.83 | 1.68 | 0.19 | 1.08 | 0.56 | 1.08 |
| Autumn | R | 4.66 | 5.59 | 3.34 | 3.15 | 8.27 | 19.3 | 0.36 | 3.69 | 0.32 | 4.80 | 2.63 | 0.55 |
|  | TSS | 0.20 | 0.42 | 0.52 | 0.10 | 1.74 | 5.49 | 0.41 | 0.15 | 0.013 | 0.56 | 0.55 | 0.56 |
|  | SPC | 0.021 | 0.026 | 0.062 | 0.015 | 0.17 | 1.06 | 0.044 | 0.022 | 0.003 | 0.054 | 0.05 | 0.054 |
|  | PP | 0.001 | 0.001 | 0.001 | 0.002 | 0.004 | 0.027 | 0.001 | 0.004 | 0.000 | 0.002 | 0.001 | 0.002 |
|  | DP | 0.007 | 0.004 | 0.002 | 0.001 | 0.021 | 0.56 | 0.016 | 0.020 | 0.001 | 0.012 | 0.009 | 0.012 |
|  | TP | 0.008 | 0.005 | 0.003 | 0.003 | 0.025 | 0.59 | 0.017 | 0.024 | 0.001 | 0.014 | 0.010 | 0.014 |
|  | PN | 0.005 | 0.003 | 0.005 | 0.000 | 0.019 | 0.17 | 0.011 | 0.022 | 0.000 | 0.011 | 0.008 | 0.011 |
|  | DN | 0.040 | 0.021 | 0.006 | 0.001 | 0.15 | 2.70 | 0.085 | 0.021 | 0.003 | 0.047 | 0.052 | 0.047 |
|  | TN | 0.045 | 0.024 | 0.011 | 0.001 | 0.17 | 2.87 | 0.095 | 0.042 | 0.003 | 0.058 | 0.057 | 0.058 |
| Winter | All | 0 | 0 | 0 | 0 | 0 | 0 | 0 | 0 | 0 | 0 | 0 | 0 |
| Year | R | 101 | 110 | 87.1 | 109 | 117 | 253 | 69.4 | 97.0 | 61.7 | 97.9 | 19.8 | 0.20 |
|  | TSS | 16.5 | 22.4 | 138 | 125 | 43.5 | 197 | 34.8 | 61.1 | 40.8 | 73.8 | 46.3 | 0.63 |
|  | SPC | 1.64 | 2.16 | 11.1 | 9.01 | 4.09 | 73.3 | 3.45 | 6.12 | 5.80 | 6.10 | 3.3 | 0.54 |
|  | PP | 0.06 | 0.05 | 0.19 | 0.22 | 0.14 | 2.38 | 0.12 | 0.13 | 0.20 | 0.13 | 0.1 | 0.50 |
|  | DP | 0.51 | 0.40 | 0.29 | 0.54 | 0.47 | 8.30 | 1.06 | 1.03 | 0.65 | 0.64 | 0.3 | 0.44 |
|  | TP | 0.56 | 0.45 | 0.47 | 0.76 | 0.61 | 10.7 | 1.17 | 1.16 | 0.85 | 0.76 | 0.3 | 0.38 |
|  | PN | 0.42 | 0.27 | 0.93 | 0.82 | 0.65 | 12.6 | 0.89 | 1.12 | 0.69 | 0.81 | 0.3 | 0.34 |
|  | DN | 4.04 | 3.03 | 2.14 | 3.47 | 3.96 | 74.9 | 8.53 | 3.13 | 3.81 | 3.74 | 1.9 | 0.52 |
|  | TN | 4.47 | 3.29 | 3.06 | 4.29 | 4.61 | 87.5 | 9.42 | 4.25 | 4.50 | 4.54 | 2.0 | 0.44 |

Figure S2. (a) spatial distribution of TSS yield and (b) spatial distribution of SPC losses during H-years 2005-2015 within the SSW

Figure S3. (a) spatial distribution of PP losses and (b) spatial distribution of DP losses during H-years 2005-2015 within the SSW

Figure S4. (a) spatial distribution of PN losses and (b) spatial distribution of DN losses during H-years 2005-2015 within the SSW
